# What does PrEP mean for ‘safe sex’ norms? A qualitative study

**DOI:** 10.1371/journal.pone.0255731

**Published:** 2021-08-05

**Authors:** Bridget Haire, Dean Murphy, Lisa Maher, Iryna Zablotska-Manos, Stephanie Vaccher, John Kaldor

**Affiliations:** 1 Kirby Institute, UNSW Sydney, Kensington, New South Wales, Australia; 2 Westmead Clinical School, University of Sydney, Westmead, New South Wales, Australia; University of Toronto, CANADA

## Abstract

While HIV pre-exposure prophylaxis (PrEP) is highly effective, it has arguably disrupted norms of ‘safe sex’ that for many years were synonymous with condom use. This qualitative study explored the culture of PrEP adoption and evolving concepts of ‘safe sex’ in Sydney, Australia, during a period of rapidly escalating access from 2015–2018, drawing on interviews with sexually active gay men (n = 31) and interviews and focus groups with key stakeholders (n = 10). Data were analysed thematically. Our results explored the decreasing centrality of condoms in risk reduction and new patterns of sexual negotiation. With regards to stigma, we found that there was arguably more stigma related to *not* taking PrEP than to taking PrEP in this sample. We also found that participants remained highly engaged with promoting the wellbeing of their communities through activities as seemingly disparate as regular STI testing, promotion of PrEP in their social circles, and contribution to research. This study has important implications for health promotion. It demonstrates how constructing PrEP as a rigid new standard to which gay men ‘should’ adhere can alienate some men and potentially create community divisions. Instead, we recommend promoting choice from a range of HIV prevention options that have both high efficacy and high acceptability.

## Introduction

HIV pre-exposure prophylaxis (PrEP) has transformed the landscape of HIV prevention. It forms part of a series of behavioural and biomedical interventions of varying levels of efficacy that have disrupted the normative power of condoms in HIV prevention discourse from the 1990s onward. Other interventions in this series have included negotiated safety [1], post-exposure prophylaxis [2], strategic positioning [3], serosorting [4] and treatment-as-prevention [5]. PrEP is highly effective at preventing HIV [6], and has the advantages of not being coitally dependant and providing receptive sexual partners with an intervention they can use without requiring the insertive partner’s cooperation [7].

Despite these advantages, the use of PrEP in populations of gay, bisexual and other men who have sex with men (GBMSM) was initially problematised by some prominent figures in the United States gay community when first approved in the US. Michael Weinstein, president of the AIDS Healthcare foundation, dismissed PrEP as a ‘party drug’; Larry Kramer, founding member of both the Gay Men’s Health Crisis and activist organisation ACT-UP, described taking a pill to prevent HIV rather than using a condom as ‘cowardly’. Freelancer David Duran wrote disapprovingly that PrEP gave ‘gay men who prefer to engage in unsafe practices’ a way to ‘bareback’ without having to worry about HIV in a piece memorably titled ‘Truvada whores?’, referencing the brand name of the medication used for PrEP [8]. (Ironically, PrEP advocates then adopted ‘Truvada whore’ as a cultural meme promoting PrEP use.) The stark community divisions between those advocating for PrEP and those warning that it could do more harm than good signal the cultural significance of condom-protected sex as normative in HIV prevention discourses for GBMSM, despite the raft of other interventions listed above that had to some extent already displaced condoms [1–4].

‘Safe sex’ (or ‘safe(r) sex) was a concept generated from the very earliest days of the HIV epidemic. The development of safe sex culture–which included, but was not confined to, condom use–focused on articulating and promulgating menus of sex practices that enabled rich expression and enjoyment of sex while precluding HIV transmission between partners. There are examples of ‘safe sex’ materials developed even prior to there being certainty that a sexually transmissible virus was the cause of AIDS [9]. Taking collective responsibility for sexual health and the avoidance of HIV transmission among gay men was described by Weeks as a concrete exercise in sexual citizenship, and he suggested that men who failed to do this risked moral pariah status [10].

For many years in Australia, the term ‘safe sex’ was synonymous with condom use, even though other forms of safe sex were articulated and practiced [1]. Maintaining high prevalence of condom use was deemed critical to controlling HIV incidence by community-based organisations and public health experts alike [11–13].

By 2010, however, there was emerging evidence of the effectiveness of new antiretroviral strategies to reduce or prevent HIV transmission to sexual partners, either by suppressing the viral loads of people living with HIV, or through the use of antiretroviral drugs as prophylaxis by HIV negative people–PrEP [14]. One of the normative challenges that PrEP brought to HIV prevention discourse was that it required individuals to acknowledge a risk (condomless or ‘bareback’ sex) that gay men had been told to avoid for three decades, outside of relationship sex [15]. Although the efficacy of treatment-as-prevention also allowed for consideration of ‘bareback’ sex, it was premised upon the use of antiretroviral drugs in people living with HIV. Suppression of the infective agent is a time-honoured strategy in infectious disease control and is less contentious in that context, though in practice some HIV negative men remain nervous despite the strong evidence of effectiveness [16]. With PrEP, the focus shifted to the routine use of antiretroviral drugs in HIV negative people potentially for protracted periods of time, an approach analogous to malaria prevention in travellers but on a far greater scale. This shift was described by Thomann as ‘the pharmaceuticalisation of the responsible sexual subject’ and is connected to ‘end of AIDS’ discourses that posit HIV prevention as a medical and technological problem [15].

Recent research has also shown both that taking PrEP is associated with lowered anxiety in gay and bisexual men who would otherwise be at risk of HIV [17–19], and that clinicians will prescribe PrEP to gay men where there is no clear clinical risk of HIV acquisition, speculating that there might be undisclosed risk factors [20].

To date there has been considerable qualitative research on the willingness of GBMSM to use PrEP, its acceptability [21–26], and community perceptions of its value in HIV prevention [27]. Research in Canada and the U.S. has also explored the impact of PrEP with respect to sexual health, communication and behaviour and social and community issues among gay and bisexual men [19, 28]. However, there has been little Australian research that explores the meaning of PrEP and how men in gay male sex cultures see it shaping evolving norms of ‘safe sex’.

This study investigated perceptions of PrEP and conceptualisations of ‘safe sex’ during the period of incrementally increasing access in Australia (2015–2018), drawing predominantly upon perspectives of GBMSM, and also on stakeholders comprising HIV community staff and healthcare providers. At the beginning of the study, PrEP was only available through very limited trials and through personal importation. Access changed dramatically in March 2016, when large-scale implementation studies commenced, with more than 10,000 GBMSM enrolled in New South Wales (NSW) [29]. In April 2018, subsidised access under Australia’s Pharmaceutical Benefits Scheme made PrEP available nationwide at a standard, subsidised price [30]. Thus, this study spanned a period of rapid change in PrEP access and uptake with data collection beginning in October 2015 continuing until December 2018. The study aimed to explore how PrEP was impacting on sex cultures–how GBMSM saw PrEP as affecting their sex practices, as well as perspectives on how PrEP affected existing cultural norms for HIV prevention.

## Methods

The Sydney In-depth PrEP study (SIn-PrEP) was a qualitative study that explored evolving norms of ‘safe sex’ during the introduction of PrEP in Australia.

SIN-PrEP drew on participatory action research methods with respect to data collection, analysis and communication of results [31]. Prior to data collection, a reference group was established to guide the research. This comprised representatives from the local LGBTIQ, HIV positive and transgender community organisations; and two researchers with extensive experience in research on gay male sexuality. This group met regularly in the early period of data collection to discuss initial findings and developments in PrEP access. As data collection progressed, the first author met periodically with representatives of the local community organisation ACON (formerly known as the AIDS Council of New South Wales), to discuss how findings could inform health promotion campaigns under development, and participated in information sessions with the community organisation to discuss implications. Study findings were reported to and discussed with community organisations prior to presentation or publication so that findings could inform development of health promotion campaigns.

### Recruitment

Data collection commenced in October 2015 and ceased in December 2018. Study participants were drawn from three distinct populations–sexually active GBMSM, clinicians involved in PrEP prescribing, staff working in HIV and LGBTIQ+ community organisations–each with different recruitment strategies. Sexually active GBMSM community participants (n = 31), (hereafter ‘gay community participants’, as these participants identified as gay) were recruited primarily through the social media channel of a local community-based LGBTQ+ organisation, ACON, supplemented by fliers distributed at gay community organisations, events, venues and word of mouth. This group included HIV negative men taking PrEP, HIV negative men who chose not to take PrEP and men living with HIV. Both cis and trans identified gay men were eligible for the study, and participants were recruited from Sydney, NSW. In 2016 and 2017, there was further targeted recruitment through Kirby Institute research data bases purposively inviting transgender gay men, and gay men on PrEP access studies who reported they had ceased taking PrEP. Only people who had given permission to be contacted for research participation opportunities were contacted using this method.

Clinicians from public sexual health clinics and general practice with high caseloads of GBMSM (n = 6) were purposively selected. Community-based staff (n = 4) were recruited through invitations to major LGBTIQ+ organisations which passed the invitations onto key personnel who then decided whether to participate.

#### Data collection

Data were collected in the form of in-depth semi-structured interviews for clinicians (n = 6) and gay community participants (n = 31), and a focus group of community-based professionals (n = 4). Interviews were audio recorded and transcribed verbatim by a professional transcriber. Interviews lasted approximately 60 minutes, while the focus group ran for 90 minutes. Interviews were usually held face-to-face, although three gay community participants were interviewed by phone. Participants in the gay community group chose their own pseudonyms. Health care providers were assigned numbers (1–6), as were focus group participants. Gay community participants were interviewed individually as they were discussing very personal issues. Data were collected from community-based professionals in a focus group, as this allowed the for a rich discussion where participants built on each other’s views and compared experiences, without privacy risks as they were not discussion their own private behaviour.

All data were collected by the first author, who is a queer-identified woman with extensive networks in the LGBTIQ+ and HIV communities.

#### Domains of interviews and focus groups

Gay community participants were asked questions about how and why they saw PrEP as relevant to their sexual lives, whether or how it was changing their sexual lives, and how they rated the importance of sex in their lives. HIV negative men were also asked about the importance of remaining HIV negative, in addition to other questions about access to PrEP and adherence for those taking PrEP. Health care providers and community-based professionals were asked about emerging issues in the provision of PrEP, their views on optimal implementation and the challenges of health communication. Community-based professionals in focus groups were asked about the impacts of PrEP of ‘safe sex’ health promotion, complexities of access and observed changes in community norms.

### Research ethics

This study was approved by the University of New South Wales Human Research Ethics Committee (approval number HC15305) and the ACON Research Ethics Review Committee (RERC 2015/08).

All participants who participated in face-to-face interviews or focus groups provided written informed consent. Participants interviewed by telephone provided formal verbal informed consent. Participants were not remunerated for their participation.

### Analysis

Transcripts from interviews and the focus group were reviewed and then coded using NVIVO (v11-12) software. Coding was initially inductive and comprised descriptive (e.g. ‘condom use–kills erection’) and conceptual codes (e.g. ‘citizenship’). Codes were reviewed and mapped in relation to each other, and developed into key themes by the first author, in discussion with reference group members, study investigators and stakeholders, and at formal presentations of preliminary findings. Descriptive themes (e.g. ‘STI testing and communication’ and ‘advocating/explaining PrEP through social media’) were further compared and analysed, leading to higher order concepts (e.g. ‘Responsibility and care’) drawing on Braun and Clarke’s six step process of reflexive thematic analysis [32, 33].

## Results

A total of 24 HIV negative gay men currently or recently on PrEP, seven gay men who had never taken PrEP (two HIV positive, five HIV negative), and six healthcare providers took part in semi-structured, in-depth interviews. One focus group was conducted with four community HIV sector staff. Two of the HIV negative men currently or recently taking PrEP were transgender and 22 were cisgender. Gay community participants were aged between 18–53 years (median 38 years; community-based staff and healthcare providers were not asked their ages).

All gay community participants described themselves as sexually active. Many had primary relationship partners or husbands, but also had other regular and/or casual partners. Among those with primary relationship partners, relationship agreements included complete openness, ‘don’t ask don’t tell’ agreements, monogamy with exceptions (such as other partners allowed when travelling), playing together (having sex with other partners together) and monogamy. This article draws predominantly on the interview data with gay community participants.

Three major cross cutting themes were identified. ‘Changing norms and clashing symbols’, encompassed the decreasing centrality of condoms in risk reduction and participants’ responses to that, and has a sub-theme on negotiation where the emergent norms are discussed in the specific context of sexual negotiation. ‘Stigma’ encompassed both stigma related to HIV and stigma related to *not* taking PrEP. ‘Responsibility and care’, comprised participants’ accounts of their views of activities as seemingly disparate as regular STI testing, promotion of PrEP and/or other risk reduction in their social circles, and contribution to research, which were nevertheless linked conceptually in participants’ discourse to ‘giving back to’ or promoting the wellbeing of their communities.

### Changing norms and clashing symbols

Participants across all three groups strongly endorsed the idea that established norms of ‘safe sex’ had changed, and that condom use was no longer central. Although most of the men in the gay community participant group had been having at least some condomless sex before PrEP, nearly all these men, whether on PrEP or not, reported that their own sexual practice had been affected directly or indirectly by increasing PrEP access. This impact was in the form of reduced condom use in casual sex. Among the sexually active men not on PrEP, there was a minority view that PrEP could not and arguably should not replace condom use, as they deemed condom use to be central to STI control. Many of the men on PrEP or those living with HIV, however, deemed curable STIs a minor annoyance only, as can be seen in the following quote.

STIs are not as of concern for me, you know. For the sake of the argument, you go in and get a jab. You go and take a couple of pills, you know, and, and we’re fine. HIV’s the big one that we don’t have a cure for. Teddie, 32, on PrEP

For many participants, a shift away from a condom-based norm while remaining protected from HIV brought a new sense of freedom, regardless of the lack of protection from other STIs.

I feel like shackles have been loosened a little. Chukki, 43, on PrEP

This freedom was connected to the physical pleasures of condomless sex, as indicated by Mannie, a 35 year old gay community participant who expressed this as “*I don’t like being fucked by a plastic bag”*.

Some men however perceived that there were socially valuable aspects of ‘condom culture’ which they feared were being lost. For these men, condom use had a symbolic value as a marker of caring either specifically for a sex partner or more broadly for ‘community’ by adopting tangible sexual practices that prevented the transmission of HIV. For men who perceived that condom use could indicate care, there was some concern that PrEP could symbolically erode this.

If someone only wants to fuck you without a condom, then are they actually thinking about the bigger consequences of the act? Steve, 53, on PrEP

Other men however used advocacy for PrEP in their virtual and real-life social circles as a way of protecting and promoting community values.

I made like some Facebook post about it … My words were: it’s a way for HIV negative people to be active in fighting HIV. Mark, 24, on PrEP

With regard to how PrEP impacted on the concept of an inclusive community, again there were clashing perspectives. HIV positive participants suggested that PrEP was diminishing what they perceived as a sexual division between HIV negative and positive men.

There’s quite a big split between condoms, people that use condoms consistently and people that use PrEP. What’s sort of happening I think is that people that are on PrEP are a lot more open to sleeping with people that are positive. Mike, 38, HIV+

There were two facets identified in this–firstly, that taking antiretroviral drugs opened HIV negative men up to understanding social issues related to taking a medication associated with HIV, and secondly, that negative men taking PrEP were less likely to serosort (proactively choose partners known or assumed to be the same serostatus) [4]. One of the HIV positive participants, however, who only had condomless sex, said that he still serosorted.

I will not choose someone that’s, that is HIV negative. [Okay] Yeah. [Yeah] I’d only, I only have sex with people that are HIV positive. Ron, 40, gay community participant, HIV+

Notably, not all HIV negative participants, whether on PrEP or not, were accepting of having known HIV positive men as sexual partners, and in particular were troubled by the idea of condomless sex with a known positive partner despite other risk-reduction interventions such as PrEP or the potential partner having an undetectable viral load.

I understand that someone who, has an undetectable viral load is, you know, safe. But, nevertheless, it just kind of plays on your mind. Josh, 45, takes PrEP periodically, such as when travelling.

One HIV negative participant not on PrEP was adamant that he would only have condomless sex with an HIV positive partner if he could see their viral load test results.

Like there’s guys I’ve met on-line who, one of them’s positive and he wants to do it without the condom. And I said, “I wanna see your [viral load] blood test [results].” Nick, 57, not on PrEP

While almost all participants were very clear that they understood that an undetectable viral load meant ‘safe sex’ from the perspective of HIV risk, several said they would expect a positive person with an undetectable viral load to use a condom. Others admitted that they avoided known HIV positive men as sex partners, though recognising that they probably had had unacknowledged HIV positive sex partners.

### Negotiation

How risk reduction was negotiated for casual sexual encounters was a major issue of debate regarding changing norms. In sexual negotiation, the massive changes caused by the increasingly pervasive role of on-line sex applications (hereafter ‘hook up apps’) was as much an issue as the changes in HIV risk reduction occasioned by PrEP and treatment-as-prevention, particularly for older men who were veterans of gay bars and sex-on-premises-venues. PrEP-taking participants were divided as to whether they would list ‘on PrEP’ on their profiles, as this set up the presumption of condomless sex–on the one hand, this was seen as increasing the attractiveness of a profile (hence increasing sexual capital), but on the other, it would shut down the potential for negotiation.

I figure that the only people who need to know that are the people who are naked next to me… if you wanna have sex with me, I actually want to have some connection with you as a human being. Steve, 53, on PrEP

Hook up apps were also a medium for discussion of PrEP—both for providing information about it to curious others, and also for heated and sometimes polarised debate about the social and community value of PrEP.

Having PrEP listed on a hook-up app was widely seen as something that forestalled negotiation about HIV risk reduction.

If you do have it on, they take that as like, “Oh, he’s going to like be into like bare-back. Like no condoms. Calvin,18, on PrEP

Another participant, who was taking PrEP but had to stop due to unmanageable side effects, noted the difference in both volume and quality of responses he got on hook up apps from when he had ‘on PrEP’ in his profile and when he subsequently removed it.

The minute you put [PrEP] out there [on your profile] people would get straight to the point with what they wanted to do with you. And like, “Oh, okay. This is kind of cool.” And then you’ll get a lot more of on-PrEP guys message you as well…. I’m like, “Whoa! Okay. No! No! Can I have a conversation with you first? See your face first? That’d be nice.” You just don’t get that [when it’s not on the profile]. Sussman, 30, former PrEP user.

For some men, particularly those who expressed some difficulty with negotiating with sex partners, PrEP was a way of protecting themselves without any need for communication about HIV risk.

Basically, I really didn’t know how to navigate conversations a lot or I just forgot about conversations in the moment. So this was something … I like to think I’m pretty organised so for me being able to do something daily is a lot easier than one thing like when you’re with somebody. Lance, 34, on PrEP

Several men in the study, including negative men not taking PrEP, talked about having condomless sex with a range of regular fuckbuddies with whom they had established trust relationships.

The people that I do have sex with without a condom who are on PrEP I know are tops. I know that they test regularly and I’ve, I had a long history with them before. Long-ish history. Max, 39, not on PrEP.

Several of the HIV negative men–both those on PrEP and those not–reported some experience of ‘vicarious PrEP’ [34] where one partner was on PrEP and the other relied on that for risk management by proxy. While several participants thought that this was an adequate strategy with known and trusted fuckbuddies, it was also strongly criticised by other participants.

Thus, while there was a consensus that the sex culture had changed particularly with respect to how sex is negotiated, there were differing views about the meaning of that change in this theme, and whether it was just about more freedom for condomless sex, or whether there was social value in the change.

### Stigma

Participants spoke about stigma in a range of different ways, and these accounts illustrated some of the many contradictions associated with the arrival of PrEP on the HIV prevention landscape. Some men described how the deliberate avoidance of men with HIV as sexual or relationship partners, which has been well documented [35], still persists even among PrEP users. Many participants also described how they either excluded–or were excluded by–other men because they were *not* using PrEP. Despite some consensus that PrEP should have contributed to reducing the serodivide between HIV positive and HIV negative men, the stigma associated with an HIV diagnosis was frequently spoken about as a primary reason for wanting to stay HIV negative, and sometimes for avoiding sex with known HIV positive partners even when on PrEP.

I do know that there’s like medication and it’s like manageable, but the stigma scares me…I think that’s part of the reason I haven’t been with an openly positive partner because I’m like even on PrEP I wouldn’t wanna take that risk. Calvin, 18, on PrEP.

Many participants perceived that with increased uptake of PrEP, many within gay male sex cultures had become less accepting of HIV negative men who opted not to take it.

I understand for some people there’s a lifestyle decision around using PrEP but it’s not for everyone and the stigma is that, if you’re against PrEP or you don’t think you need to take it up, that you’re somehow an idiot. So that’s the new stigma in the community. That, if you’re on PrEP, you’re a responsible, socially considerate, golden gay. And, if you’re not on it, you’re somebody who can be poo-hooed and dismissed, and attacked. Justin, 40, not on PrEP.

This idea that not using PrEP and wanting condom-protected sex diminished sexual capital was echoed across the different groups of participants. Some participants openly acknowledged that they would reject a potential sex partner if he wanted to use a condom.

If I’m at a sex party … if I turn around [and] somebody’s put a condom on, I will roll my eyes and get up, and walk away. Jack, 39, on PrEP

Jack’s reported actions convey not just a ‘no, thank you’ to prospective partner, but a pointed act of rejection. Other participants reported filtering out prospective partners who wanted to use condoms by positively selecting partners on the basis of PrEP use.

What’s your name? Are you on PrEP? Marc, 32, gay community participant on PrEP.

Other gay community participants confirmed that expressing an interest in using condoms was likely to result in rejection.

To be honest with you, if it’s in Sydney or Melbourne, you could almost guarantee that a condom’s gonna be a deal-breaker for the other person. David, 40, on PrEP.

This perception that wanting to continue to use condoms could adversely affect a man’s sexual capital was also predicted by one of the health providers.

The sexual, social milieu is going to change and, if you want to have sex, you’re going to have to adapt to the new flavour. Unless you’re the cutest boy on earth, negotiating condom use is going to become harder. Healthcare provider #3.

HIV community professionals working in a community-based HIV testing site also noted that some men who had previously been condoms users were turning to PrEP due to peer pressure:

These days I’m seeing more and more people come with, have been using condoms until today but they find that they, when meeting people who are on PrEP and they don’t want to use condoms, they find that conversation a bit of an issue. So eventually they feel like they are missing out because the guy on PrEP ends up not necessarily having sex with them because they don’t want to use a condom. So some people have decided to go on PrEP because they find that their casual partners don’t want to have sex with them ‘cause they won’t use a condom. HIV community professional #4

In this thematic area, there was little evidence of PrEP use or PrEP users being shamed or stigmatised; rather it was men who chose not to use PrEP who reported feeling that their social and sexual capital was diminished. Regarding HIV stigma, many participants accepted that it was a given. While some reflected on how their PrEP use could potentially reduce HIV stigma, one of the key reasons that HIV negative participants gave for wanting to remain HIV negative was to avoid the perceived social burden and loss of sexual capital attached to an HIV positive diagnosis.

### Responsibility and care

From a range of domains including condom use, sexually transmissible infections (STI), testing, and participating in research, we identified the cross-cutting theme of responsibility and care. That is, participants framed their responses on these issues in terms of either interpersonal responsibility or responsibility at a broader social level. Several participants framed frequent STI testing and subsequent communication of positive results to partners as a considered strategy of “*stopping the spread of them as much as I can”* ***(***Jack, 39, PrEP). This strategy included testing more regularly than the recommended three months, and testing after significant risk events (such as after a sex party of 20, as cited by one participant). For some participants, this sense of responsibility also extended to wanting to ensure that their sex partners had the skills to reduce their HIV risk. For one participant on PrEP, this meant resisting partners who wanted to rely on vicarious PrEP (that is, assuming that condomless sex is safe because a partner is on PrEP, when not on it oneself).

I think you have a moral responsibility to ensure that the person you’re actually having sex with is–if you actually have some knowledge and some ability to prevent that person from catching HIV, then, then you need to reinforce it in some sort of way and that’s either condoms or PrEP. And, if you can’t have the discussion and know that person’s gonna be on PrEP in the near future, then you need to reinforce with the condoms. Gordon, 53, on PrEP

Two other participants talked at length about how they promoted regular STI and HIV testing in their social circles, particularly to younger friends.

I spend a lot of time just checking in on my friends… “Hi, how are you? … Hey, have you had your tests recently? Mannie, 35, on PrEP.

Several participants talked about the importance of PrEP being available for men in serodiscordant relationships, even if the HIV positive partner had an undetectable viral load and the couple was monogamous, meaning that there was no HIV transmission risk. The rationale for this was so that the HIV negative partner was taking responsibility for his own safety, not relying on his partner’s adherence to medication to manage HIV risk.

It may be doubling-up but then it gives the person capacity to, to be responsible for their own safety. Josh, 45, taking PrEP periodically

In addition to wanting to take responsibility for their own sexual health, there was also an element of distrust of a partner’s undetectable viral load as being a reliable form of safe sex. As noted earlier, some participants voiced nervousness of condom-free sex with known positive partners.

Many men also talked about responsibility in terms of their participation in research to generate data for the good of the community.

One of the reasons I’m happy to do this [interview] however long this takes out of the day is I just think it’s a very good thing. [PrEP] has been very good for me and, if I can do things that encourage it to be more readily available and more accessible, I’m happy to do that. Ian, 53, on PrEP

The concept of being a responsible sexual subject was important to the gay community participants in this study, regardless of whether they were HIV negative or positive and whether or not they took PrEP. While for some condoms remained important both practically and symbolically, others were actively reframing practices such as STI testing as ways of taking responsibility. This concept of research participation as a way of enacting a responsible attitude to community was also raised repeatedly by participants–this was not related to a question asked by the interviewer but volunteered spontaneously by several participants.

## Discussion

This study explored the impact of PrEP on evolving gay male sex cultures focusing on the perceptions of gay men in Sydney, Australia, and included perspectives from health service providers and community-based stakeholders. The findings reflect that the meaning of PrEP in the lives of these men needs to be understood in the context of sex cultures deeply inflected with norms that arose in response to the risk of HIV. Taking PrEP can provide access to the pleasure of condomless sex without HIV risk, but it also disrupts decades of community norms where practices of risk reduction–condom use, serosorting [4], negotiated safety [1], strategic positioning [3]–all required negotiation and had to some degree become associated with a demonstration of care for self and other, sometimes described as ‘sexual citizenship’ [10]. The displacement of older ‘safe sex’ norms did not, however, indicate that participants were less invested in community. Many of the PrEP-taking men in this study talked about how other practices related to PrEP such as frequent STI testing and proactive partner notification of diagnoses, advocating for and educating others on PrEP, and participating in research could also be construed as acts of care for partners and community [36], or a new form of ‘citizenship’.

In considering the impacts of PrEP uptake on the sexual culture, we explored how discourses about PrEP contributed to shaping a normative goal of a new ‘safe sex’ culture that embraces a much broader menu of options [37]. We contend that the aspirational social norms articulated by the participants and discussed herein comprise a sex culture in which risks are minimised, participants have a fair chance of finding sexual satisfaction regardless of HIV serostatus or choice of HIV risk reduction intervention, free from stigma and discrimination, with community practices that sustain and promulgate these norms. In each of these three areas–minimising risk, having discrimination-free satisfying sex, and developing and sustaining community practices that support these norms–there were areas of contention.

Nearly all the gay community participants reported that their own sexual practice had changed with increasing community uptake of PrEP, in that they were less likely to use condoms in casual sex. This echoes findings of Newman et al and Pantalone et al [19, 28] but contrasts with a 2017 U.S. study that found that participants reported that while PrEP brought a feeling of relief or reprieve from HIV stress, it did not directly impact their practice [38]. The difference with the 2017 study may reflect increasing community confidence with the effectiveness of PrEP.

Confidence in PrEP did not, however, necessarily mean that participants were comfortable having sex with known HIV positive partners. While some participants–particularly those in serodiscordant relationships–were very clear that such sex would be ‘safe’, others expressed avoidance of sex with known positive partners despite taking PrEP. These participants themselves recognised this avoidance as irrational, given that the point of PrEP is to prevent HIV acquisition and that they had likely had sex with undisclosed HIV positive partners. Thus, while some of the HIV positive men saw PrEP use as dissolving some of the barriers to sex between people of different serostatus–‘bridging the serodivide’ [39], some HIV negative men continued to have discriminatory attitudes towards known HIV positive partners. This contrasts with results from two separate U.S. based studies [18, 28], which both found that PrEP uptake helped to diminish feelings of stigma toward men with HIV. Again., this difference may be due to increased confidence with PrEP efficacy, as the U.S studies recruited later than our cohort.

Within our cohort, there was also evidence of a significant bias against men who opted to use condoms as their primary risk reduction method, echoing findings of both Newman et al and Pantalone et al, who noted increased pressures for condomless sex and increased challenges in negotiating condom use [19, 28] This finding in three separate studies leads to a disquieting conclusion that opting to use condoms as primary risk reduction and/or a making a disclosure of HIV positive status, could diminish an individual’s sexual capital and limit opportunities for satisfying sex.

With regard to supportive community practices that respect diversities and different choices, some men saw the combination of PrEP and hook-up apps as decentring communication around sexual practice and eroding the community building that some associated with sexual negotiation around condom use. Nevertheless, they reported enjoying the sexual freedoms afforded by PrEP.

The finding that non-use of PrEP could be stigmatised was also seen in a Canadian study [40]. Orne and Gall used a model of ‘PrEP citizenship’ to explain how widespread PrEP uptake produced a culture of conformity to PrEP-centred regimens. This model included taking up PrEP (‘conversion’), advocating it to others (‘evangelising’) adherence, (‘self-governance’) repeat testing (‘surveillance’), and posited non-users as ‘potentially infectious’ and ‘stigmatised and irresponsible people’ (p. 657) as distinct from the ‘good citizens’ taking PrEP. This model has parallels with Thomann’s neoliberal sexual subject who acknowledges HIV risk [41], takes pre-emptive pharmaceutical action against it, and becomes ‘biomedically responsibilised’. Both Thomann’s and Orne and Gall’s analyses foregrounded how ‘PrEP advocacy’ or ‘demand creation’–as distinct from advocacy for a choice of HIV prevention interventions available to all–can marginalise those who make different choices, such as the choice to use condoms. Evidence from this study supports that contention, in that some participants took up both PrEP use and PrEP advocacy as ‘the’ response to HIV prevention, which alienated men who did not want to take antiretrovirals preventatively. Of note, however, some PrEP takers in this study resisted discourses of conformity to universal PrEP use and continued a champion a range of options depending on circumstances. In particular, some participants discussed PrEP use in the context of travel as distinct from during everyday life, given that for some travel was an opportunity for non-relationship sex including within the context of a relationship agreement. This phenomenon further breaks down the binary of ‘PrEP user’ and ‘non-user’ [19], and documents a new form of risk-reduction adaptation.

The qualitative approach of this study enabled a rich and nuanced analysis of the evolution of safe sex norms concomitant with the advent of PrEP. While the specific impacts of PrEP on HIV risk reduction practice was one focus, our other focus on normativity within these sex cultures illuminated how care can be demonstrated between casual sex partners when the problem of HIV risk has been largely dealt with by a daily pill, and how differences in values could or should be accommodated in a sex culture that aspires to not discriminate on the basis of serostatus or choice of HIV risk reduction method.

PrEP access in Australia was at least four years behind the U.S. approval in 2012, as the first large scale implementation study in Australia began in 2016 [29] and subsidised national access began in 2018 [30]. This time lag between Australia and the U.S.–and the fact that Australian community-based HIV organisations had to work hard to achieve subsidised access [42]–may in part explain why there was a less severe anti-PrEP backlash once the intervention was available. The Australian HIV community sector, health care providers and sexually active gay men had seen the ‘Truvada whore’ controversy [8]–which stereotyped PrEP users as promiscuous and irresponsible–play out in the U.S. before PrEP was widely available. The context of having no nationally accessible, funded mechanisms for PrEP access in Australia some four years after the FDA approval arguably contributed to heightening pro-PrEP sentiment [41], because the global connectedness of gay male communities allowed men in Australia to witness the sexual freedom that PrEP facilitated in the U.S. and recognise the advantages it could bring.

This study has some limitations. Gay community participants had to contact the researchers to take part in the study, so those with strong views on the impacts of PrEP may have been more likely to volunteer. The majority of participants were white, but we did not collect data systematically on ethnicity. Accordingly, the study may overrepresent the views of white gay men. Data were also collected over a period of three years during a period of rapid change, so are not a snapshot of a point in time, but a collection of perspectives that were in the process of evolution. Most of the study participants were taking PrEP, and a significantly smaller number of HIV negative men not on PrEP and HIV positive men were included, so while the sample includes perspectives from a range of different actors, they are not equally sampled. Finally, as this paper is about the impacts of PrEP on a sex culture, the voices of the gay community participants have been privileged over those of the healthcare providers and HIV community-based professionals.

## Conclusion

The impacts of PrEP are complex and need to be considered in the context of evolving gay male sex cultures in which PrEP is only one element. PrEP was not the catalyst for condomless sex for most of the men in this group, but the introduction and scale-up of PrEP access arguably enabled men to talk about condomless sex more openly, and to consider what matters in gay male sex cultures where condom use is decentred. This study has important implications for health promotion. It reveals how new community conversations about HIV prevention can promote PrEP use as the single best option, constructing it as a rigid new standard to which men ‘should’ adhere, instead of promoting and promulgating choice and genuine acceptance that different values can mean that different options may work better for some individuals. The identification of a potentially damaging emerging norm in these data, that of PrEP use as being positioned prescriptively as the ‘best’ form of HIV prevention for HIV negative men and stigma attaching to non-use, informed the development of ACON’s 2017 campaign ‘How do you do it?, in which the importance of individual choice from a range of effective options was emphasised with respect to HIV prevention [43].

While recognising the great importance of PrEP for many men, this study suggests that, rather than promoting PrEP as the new ‘safe sex’ orthodoxy, there is a need to ensure that there is a range of HIV prevention options that have both high efficacy and high acceptability. Accordingly, health promotion should focus on building community attitudes that respect diversity and challenge the primacy of any one prevention tool.
